# A comparative study of skin transcriptomes and histological observations for black and white hair colors of giant panda

**DOI:** 10.3389/fmed.2022.983992

**Published:** 2022-11-24

**Authors:** Yingyun Wang, Shangyue Liang, Shaotong Tu, Zhangqi Shen, Yanjun Dong, Gang Liu, Hao Shi, Yipeng Jin

**Affiliations:** College of Veterinary Medicine, China Agricultural University, Beijing, China

**Keywords:** giant panda, histology, skin, transcriptome sequencing, KEGG

## Abstract

The Giant pandas (*Ailuropoda melanoleuca*) are mammals belonging to the bear family, order Carnivora, and their characteristic hair color and distribution has been in the spotlight. In recent years, the gradual prevalence of skin diseases in giant pandas and even the discovery of albino individuals have made the study of the substrate of their skin hair distribution more and more urgent. In this study, by comparing the skin histology and transcriptomes for hairs of different color of giant pandas, we found that the melanin contents of hair follicles at the bases of black and white hairs differed, but the hair follicles at the base of white hairs also contained some amount of melanin. The transcriptome sequencing results showed that there were great differences in the expression of the transcriptome of the skin under different hair color blocks, in which the number of differentially expressed genes in the white skin was much smaller than that in the black skin. Transcriptomes for skin tissue samples for different hair colors revealed several enriched Kyoto encyclopedia of genes (KEGG) pathways that include tumor, cell adhesion and melanocyte growth-related signaling pathways. This study provides a theoretical basis for subsequent studies on hair color distribution and skin diseases in giant pandas.

## Introduction

Giant panda (*Ailuropoda melanoleuca*) is a mammal belonging to Carnivora Ursidae (1). It originated in southern China and is famous for its round black hair around the eyes and black and white hair on the trunk and limbs. Although it has a short digestive tract of Carnivora, 99% of its diet is made up of bamboo and bamboo shoots (2). Wild giant panda (WGP) has always been the flagship species of wild animals in China due to its small number (3), and its survival has always been the focus of attention.

In recent years, the incidence of skin diseases in giant pandas has gradually increased. For example, in 2016, several adult giant pandas and cubs at Chengdu Giant Panda Conservation and Research Center appeared to exhibit black eye circles with significant hair loss and whitening, accompanied by itching (Supplementary Figure 1). After targeted treatment, the health conditions of many giant pandas were not improved and they developed chronic manifestations: total hair loss and “black eye circles” basically turned into “white eye circles” (4). These skin problems are related to the pathological changes of black hair falling or whitening around the eyes, which are called “white eye syndrome” of giant panda. And in May 2019, Wolong National Nature Reserve Administration of Sichuan Province released a picture of the world’s first white giant panda (Supplementary Figure 2). The picture clearly shows that the giant panda has white hair, white claws and red eyes. According to these external features, this particular giant panda can be judged to be an albino individual. All of the above-mentioned giant pandas showed changes in hair color, but the reasons for their changes are not clear.

Because is difficult to collect tissue samples from live giant pandas, the pathogenesis of giant panda skin disease, hair color changes and hair color formation are poorly understood. Mammalian hair color is a polygenic trait that may be influenced by the interaction of different kinds of polygenes (5). The color is usually determined by the amounts of eumelanin and brown melanin, which are synthesized by melanocytes located in the hair matrix above the dermal papilla (6). Melanocytes are found in hair follicles and in the basal skin layer. Apart from that, various studies have suggested that different body areas may have different functional colors. For example, some research teams believed that dark fur around the eyes can reduce the glare of the sun (7).

Therefore, this study used hematoxylin and eosin (H and E) staining and Illumina sequencing technology to investigate the differences and similarities in melanin content and transcriptome expression in the skin follicles under black and white hairs of WGPs, which can’t only elucidate the causes of giant panda hair color formation at the histological and genetic levels, but also explore the physiological functions of the skin tissues under different hair colors. In addition, this study can provide a theoretical basis for subsequent research on giant panda skin-related diseases and promote the development of giant panda molecular genetics research.

## Materials and methods

### Giant panda skin tissue sample collections

The study protocol was approved by the Administration of Shaanxi Foping National Nature Reserve Administration, the Scientific Committee, and the Institutional Animal Care and Use Committee.

Skin tissues from two giant pandas (one male and one female) that died from acute falls by accidentally was used in the study, and the samples were kindly donated by the Foping National Nature Reserve, China (Supplementary Table 1). After collection, one part of the skin samples was fixed with 40% formaldehyde solution for H and E staining, and the other part was immediately placed in a 5 ml sterilized EP tube equipped with RNA Keeper and stored at −20°C until RNA extraction.

### Major experimental reagents

Formaldehyde solution [Beijing Institute for Cancer Research (BICR), Beijing, China]; Potassium dichromate (Sinopharm Chemical Reagent Co., Ltd, Shanghai, China); Acid reddish (BICR, Beijing, China); Aniline Blue (Sinopharm Chemical Reagent Co., Ltd, Shanghai, China); Hematoxylin (BICR, Beijing, China); eosin (BICR, Beijing, China); Trizol (TransGen Biotech, Beijing, China); Chloroform (Tianjin Guangcheng Chemical Reagent Co., Ltd. Tianjin, China); Anhydrous ethanol (BICR, Beijing, China); Isopropyl alcohol (Tianjin Guangcheng Chemical Reagent Co., Ltd. Tianjin, China); NaOH (Sinopharm Chemical Reagent Co., Ltd, Shanghai, China); Boric acid (BICR, Beijing, China); Agarose (Thermo Fisher Scientific, Beijing, China); RNase-free water (TransGen Biotech, Beijing, China); and RNA Keeper (GENEWIZ, Suzhou, China).

### Agarose gel and solution preparations

#### 1% agarose gel

Add 1 g of agarose to 100 mL of 1 × Tris-acetate-ethylenediaminetetraacetic acid (TAE), microwave to dissolve until the liquid phase is homogeneous, allow to calm to about 60°C at room temperature, and introduce into the mold.

#### Phosphate-buffered saline buffer

Separately weigh 8 g of NaCl, 0.2 g of KCl, 0.24 g of KH_2_PO_4_ and 3.63 g of Na_2_HPO_4_-12H_2_O, add MilliQ, dissolve and volume to 1 L, autoclave and store at 4°C.

### Paraffin section preparation and hematoxylin and eosin staining of giant panda skin tissues

For histological studies, giant panda skin tissue was fixed in 10% formalin, embedded in paraffin, sectioned, and stained with H and E. The sections were subsequently observed under a light microscope, and the similarities and differences in the hair follicles within the skin at the base of black and white hairs were recorded under low and high magnification, respectively.

### Sequencing of the eukaryotic transcriptomes of the skin tissues under the black and white hairs of the giant panda

The process for eukaryotic reference transcriptome high-throughput sequencing comparisons is outlined below (Novogene, Beijing, China).

#### RNA extraction and quality control

Total RNA was extracted using Trizol (TransGen Biotech, Beijing, China) according to the manufacturer protocol. The quality, purity and concentration of total RNA were quantified using 1% agarose gel electrophoresis assay and NanoDrop 2000C (NanoDrop, USA).

#### Library construction and inspection

The mRNA was enriched by magnetic beads with Oligo (dT) (Thermo Fisher Scientific, Beijing, China). Fragment buffer was added to the purified mRNA to cleave it into short fragments, and the first-strand cDNA was synthesized with six-base random primers. Second strand cDNA was synthesized by adding dNTPs, DNA polymerase I and buffer. Subsequently, AMPure XP system (Beckman Coulter, Beverly, MA, USA) was used to purify the double-stranded cDNA. And the appropriate fragment size was selected to construct the cDNA library.

After the library construction was completed, one microgram total RNA of each sample was used as the input material. Then we used Qubit 2.0 and Agilent 2100 systems to determine the library concentration and library insert size.

#### Illumina sequencing

The library preparations were sequenced on an Illumina Hiseq 2500 platform and 150 bp paired-end reads were generated.

To ensure the quality of information analysis, the raw data (raw sequencing reads) files in FASTQ format must be filtered to obtain clean reads, and subsequent analysis is based on clean reads. The steps for raw data processing were as follows: (1) removing sequence reads with adapter. The RNA-seq adapter information can be found in the Oligonucleotide sequences for TruSeq™ RNA and DNA Sample Prep Kits manufacturer’s instructions, including RNA 5′ Adapter (RA5), part #15013205: 5′-AATGATACGGCGACCACCGAGATCTACACTCTTTCCCTA CACGACGCTCTTCCGATCT-3′, RNA 3′ Adapter (RA3), part #15013207: 5′-GATCGGAAGAGCACACGTCTGAACTCCAG TCACATCTCGTATGCCGTCTTCTGCTTG-3′. (2) Removing sequencing reads that contain ploy-N sequences with an uncertain base ratio of more than 10%. (3) Removing sequencing reads with low-quality reads (reads with Q phred <20 bases accounting for more than 50% of total read length).

#### Read mapping, transcriptome assembly, and expression level quantification

Clean reads were then mapped to the reference genome sequence (ASM200744v2) (8). HISAT2v2.0.4 software was used to map the reference genome (9). The genomic mapping result of all the sequenced reads data was pooled together and assembled using Cufflinks v2.1.1, and then compared with the giant panda reference genome using Cuffcompare.

Then the expressed values of each sample were calculated by HTSeq (the model of UNION) based on fragments per kilobase of exon model per million mapped reads (FPKM) methods (10). Pearson correlation analyses of the correlation of gene expression in all samples were performed to verify the credibility and repeatability of this experiment.

#### Differential gene expression analysis of skin tissues for white and black hairs

The input data for differential gene expression analysis was the readcount data obtained from the gene expression level analysis, the analysis was divided into three main parts (a) normalization, (b) the calculation of the probability of hypothesis testing (*p*-value) according to the model, and (c) the calculation for multiplicity-corrected *p*-value, namely, the false discovery rate (FDR), also known as *q*-value.

To screen for genes that were differentially expressed between tissues, DEGSeq v 1.12.0 and DESeq v1.10.1 were used. The resulting *p*-values were adjusted using Benjamini and Hochberg’s approach for controlling the FDR (11).

#### Gene ontology and Kyoto encyclopedia of genes enrichment analysis of differentially expressed genes

Gene ontology enrichment analysis of DEGs was implemented using the Bioconductor package GOseq (Release2.12 version), (12) and this method was based on Wallenius non-central hypergeometric distribution. KEGG Orthology Based Annotation System (KOBAS) v2.0 software was used to perform the statistical enrichment of DEGs against the KEGG pathway database, the *p*-value < 0.05 and | log2FoldChange| > 2 were considered significantly enriched by DEGs.

## Results

### Histological observation of the hair base

**FIGURE 1:**
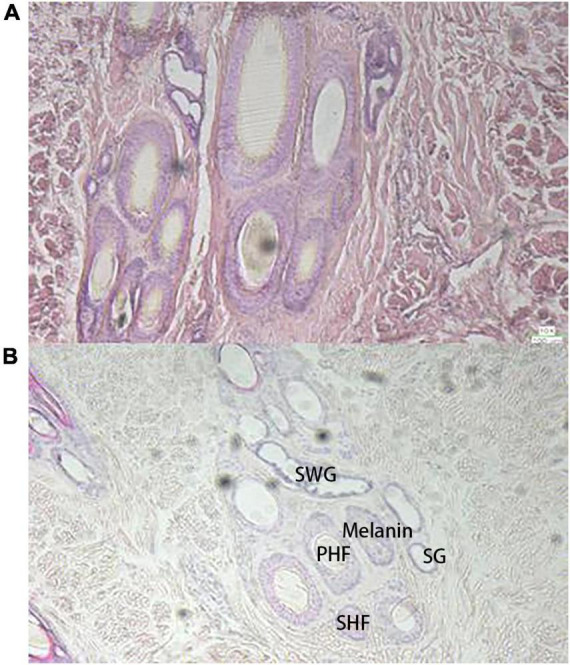
Observation of skin tissue with H and E staining sections (10×), **(A)** the black hair base; **(B)** the white hair base.

Similar to the raccoon study (13), the basal follicles underneath the black hair contained more melanin than the follicles underneath the white hair, but the skin underneath the white hair also contained some amount of melanin (Figure 1). There were also differences in melanin distribution between different black hair follicles. The basal skin beneath the white hair contained more irregularly shaped glands, including the secretory sweat gland (SWG), which was cystically distributed around the primary hair follicles (PHF), and the sebaceous gland (SG), which is smaller and more circular around the follicle. Both black and white hairs are composed of thicker bristles surrounded by finer tufts of villi.

### Transcriptome differential analysis

In this study, 4 sequencing libraries were constructed from two giant pandas, namely WGP_b1, WGP_b2, WGP_w1, and WGP_w2 (Supplementary Table 2). All the error rates of per base sequencing were lower than 0.03%, and the Phred score Q30 value of all reads was greater than 89.01%. An average of 44.52 million clean reads per sample were successfully mapped to the giant panda reference genome, and approximately, 42.80 million reads per sample were uniquely mapped among the total mapped reads (Supplementary Table 3).

**FIGURE 2:**
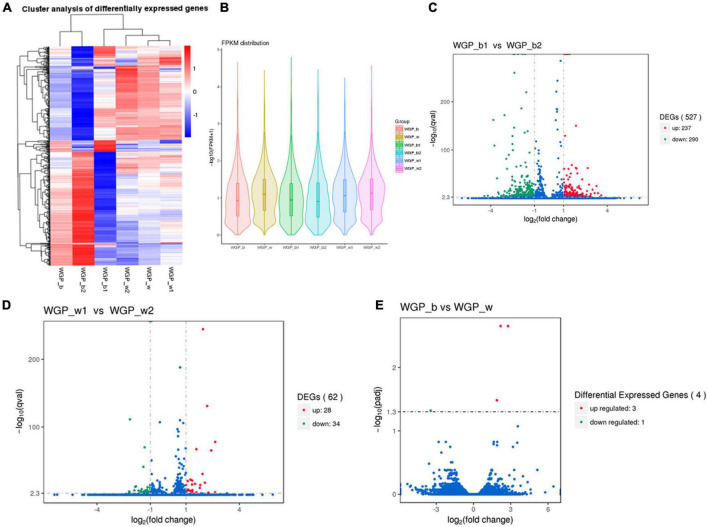
Comparison of gene expression levels. **(A)** The overall FPKM hierarchical clustering diagram, the log10 (FPKM + 1) values were scaled number and clustered. Red represents highly expressed genes and blue represents low expressed genes. The color ranges from red to blue, indicating log10 (FPKM + 1) from large to small. **(B)** FPKM violin plot with sample name on the *X*-axis and log10 (FPKM + 1) on the *Y*-axis. Each region’s Violin plot corresponds to five statistics (maximum, upper quartile, median, lower quartile and minimum, respectively, from top to bottom). The width of each violin represents how many genes were under that expression. **(C–E)** Volcano plot of the overall distribution of differential genes. Genes with significant differentially expressed genes are represented by red dots (up-regulated) and green dots (down-regulated), while genes without significant differentially expressed genes are represented by blue dots. The abscissa represents the fold change of gene expression in different samples. The ordinate represents the statistical significance of the difference in gene expression.

The FPKM distributions of all genes and violin plots were used to compare the gene expression levels in skin under different hair colors. The results showed that there is a significant difference in expression between the lower quartile and the minima when comparing black and white hairs longitudinally, the gene expression in the black under-hair skin was much higher than that in white hairs (Figures 2A,B).

Transcriptome expression analysis of two different regions of skin under white hair showed 26 groups with up-regulated expression and 34 groups with down-regulated expression. Transcriptome analysis of two different regions of skin under black hair showed 237 groups with up-regulated expression and 290 groups with down-regulated expression. While comparison of expression levels between black and white under-hair skin showed that only four groups of genes showed differences (Figures 2C–E).

### Gene ontology terms and Kyoto encyclopedia of genes pathway analysis of differentially expressed genes

**FIGURE 3:**
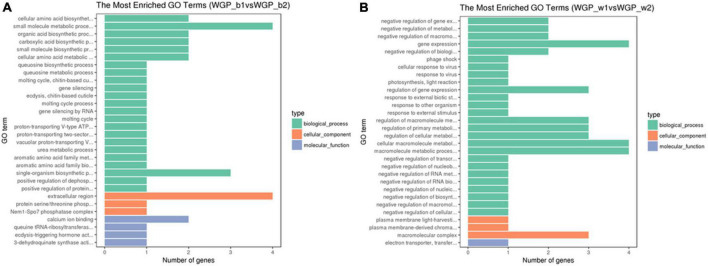
Gene ontology (GO) terms of DEGs enrichment graph. The ordinate is the enriched GO terms, and the abscissa is the number of differential genes in this term. Green is represent biological processes, orange is represent cellular components, and blue is represent molecular functions. **(A)** The Most Enrichment GO Terms in WGP_b1 vs WGP_b2, **(B)** The Most Enrichment GO Terms in WGP_w1 vs WGP_w2.

Using the above tissue-specific DEGs, the GO enrichment analyses were performed. GO terms that were significantly enriched in WGP_b1 compared to –b2 included small molecule metabolic process, single-organism biosynthetic process and extracellular region, etc. (Figure 3A). In WGP_w1 and WGP_w2, the most enriched GO terms were gene expression, regulation of cellular metabolic process, macromolecule metabolic process and macromolecular complex (Figure 3B).

**FIGURE 4:**
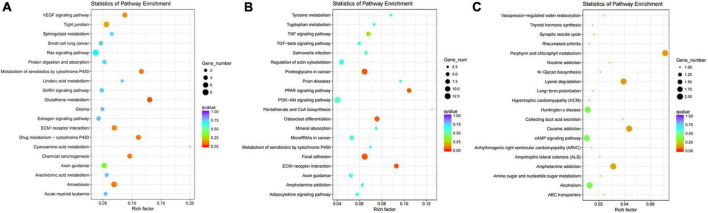
Kyoto encyclopedia of genes (KEGG) pathways enrichment scatter plot of DEGs. **(A,B)** The KEGG pathways of WGP_b1 to WGP_b2. **(C)** The KEGG pathways of WGP_w1 to WGP_w2. The vertical axis represents pathway name, and the horizontal axis represents Rich factor. The size of dots represents the number of differentially expressed genes in this pathway, and the color of dots corresponds to different *Q*-value ranges. The redder the color, the more significant the difference.

In the analysis of KEGG pathways of DEGs, we found different regions of skin under black hair showed great differences in Focal adhesion, Tyrosine metabolism, PI3K-AKT signaling pathway, Proteoglycans in cancer, TNF signaling pathway, MicroRNAs in cancer, etc. (Figures 4A,B). Among the above mentioned signaling pathways, FGF, Hedgehog, TNF, Notch and Wnt have been shown to be closely associated with hair follicle color formation. The different regions of the skin under black hair also had differential enrichment (2–8) of KEGG signaling pathways in the VEGF signaling pathway, glutathione metabolism, amoebiasis, etc. In addition, WGP_w1 and WGP_w2 (scatter plot of KEGG enrichment within the white under-hair skin group) showed that the number of differentially enriched genes in different regions of the white hair base skin was low, such as lysine degradation, porphyrin and chlorophyll metabolism, cocaine addiction and amphetamine lateral sclerosis (ALS) (Figure 4C).

## Discussion

In 2009, researchers sequenced the giant panda genome using the Illumina platform and learned that the genome contains 20 pairs of autosomes and 1 pair of sex chromosomes (14). However, due to the sample size, the genetic profile of the giant panda is still not well annotated, and there are still many unknown genes and unidentified functional fragments in the panda chromosome, the distinctive black and white markings of giant pandas and the increasing occurrence of clinical conditions such as hair loss and even albinism still do not have a clear basic understanding. In this study, we investigated the histology and transcriptomes of the skin samples under black and white hairs to provide a basic portrayal of the hair and skin of the giant panda in a healthy state by using H and E staining and the RNA high-throughput sequencing technology, and help us to understand the molecular mechanisms of the genetic regulation of hair color formation.

In skin sections, PHF and secondary hair follicles (SHF) were observed on the dorsal skin of giant pandas, and the two were mixed and clustered together in a complex hair follicle, similar in composition to that of dogs. In WGPs, the PHF is about twice the size of the SHF, and the PHF of wild-type pandas is usually surrounded by 4–5 SHF, which also contain more melanin than SHF. The basal follicles of black hairs contain more melanin particles, and the basal follicles of white hairs also contain a certain amount of melanin, which is a larger finding. The PHF was surrounded by other functional structures such as SWGs, SGs, and erector muscle. The SHF is surrounded by only a few underdeveloped SGs. According to the anatomy of the panda (15), the SGs of the giant panda are branched vesicular glands, and the dorsal and ventral SGs are underdeveloped, forming only a few vesicles around the hair follicles, which are small. In contrast, the SWGs of giant pandas are mostly apocrine, located next to the hair follicles, and have both vesicular and tubular glands. In this study, it was found that there were more irregularly shaped SWGs around the PHF at the base of white hair than black hair follicles, and the melanin content was less than that of black hair follicles. As we all know, black and white hairs play a completely different role in the way light maintains body temperature due to their different UV transmission rates: white hairs allow a large amount of UV light to reach the skin through the hairs because of their weak absorption of a certain wavelength of UV light. Therefore, all other things being equal, the skin under white hair has to face a higher heat exposure when the UV light is strong. So it is reasonable to assume that giant pandas use glands to stabilize and excrete waste, and the presence of a large number of glands around the skin follicles at the base of the white hairs would be a reasonable explanation. Of course, current studies have not addressed the presence and function of glands in giant panda skin, so it is not possible to determine exactly what kind of glands are present.

Transcriptome analysis showed significant differences in gene expression in the skin under different hair, with the number of gene copies in the skin under black hair being approximately 10 times higher than the number under white hair. Different regions of skin under black hair showed dramatic differences in gene expression compared with white hair. The DEGs of skin under white hair showed only some disease-related genes, porphyrin and lysine related pathways were differentially enriched in the KEGG pathway. In recent years, various studies have suggested that the color of different body regions of giant pandas may have different functions. Studies using big data analysis to analyze the physiological function of the black and white areas of the giant panda to distinguish hair color have been able to confirm four existing ideas to explain its unique coloring (16): The hair of the species is selectively adapted to release a belligerent message to other animals; the white hair of the giant panda is designed to better hide against the natural backdrop of the snow-capped landscape (17); the black hair of the giant panda is used to maximize heat retention in cold environments (18); Specific markings on the giant panda’s face (black eyes) are used for intraspecific communication (19). Combined with our finding that WGPs part of what we consider black hair appears brown or light brown, we hypothesize that the skin under black hair of giant pandas bears more biological functions compared with the skin under white hair, and the biological functions of the black hairs in different regions are different. However, due to the difficulty of obtaining samples, we only used samples from the back and shoulders of two giant pandas for comparison, and the results may be idiosyncratic, and more sample comparisons are needed subsequently to further confirm this result.

The transcriptomic KEGG differential enrichment analysis of skin with different hair follicle colors showed that there were differential enrichment in Tyr, EDA/EDAR, ECM-receptor interaction, WNT, TGF-activity, R, color and Notch signaling pathways, which indicated that the factors of the above signaling pathways were shuttled between the various cell types and the skin of the black and white hair base, forming a complex protein regulatory network. The differential expression of these signaling pathways and factors ultimately results in a specific black and white outer hair. The ECM-receptor interaction pathway affects the physiological activity of cell proliferation and migration, which is very important for hair follicle development; TNF and TGF-activation pathways are not only involved in tumor formation, but also have a great impact on the development of hair shaft keratin. The differential enrichment of Tyr-related pathways is a direct indication of the differential expression of tyrosinase and other related melanin synthesis factors in the skin under different hair colors, and this part of the difference is a direct factor of different hair colors. The cross-sectional comparison between the black and white hair base skin groups was not significant because the number of gene copies in the white group was too small, and the analysis of these results showed that the annotation of the giant panda gene book is still incomplete, and there are many unknown functions and uses of the variable shear products, which can lead to large errors. Therefore, it is necessary to perform more sequencing analyses, enrich the sample size, perform protein interaction studies on the enriched products, and discover more interesting new genes, which is also the direction of this study.

## Data availability statement

The datasets presented in this study can be found in online repositories. The names of the repository/repositories and accession number(s) can be found in the article/Supplementary material.

## Ethics statement

Ethical review and approval was not required for the animal study because Live animals are not used in this study, and the materials were donated by the Foping National Nature Reserve, China.

## Author contributions

YJ and HS contributed to the conception of the study. YW and SL performed the experiment. YW and ST significantly contributed to the analysis and manuscript preparation. ZS, GL, and YD performed the data analyses. All authors listed have made a substantial, direct, and intellectual contribution to the work, and approved it for publication.
