# Supplementary material for: A comparative study of skin transcriptomes and histological observations for black and white hair colors of giant panda
Source: Front Med (Lausanne). 2022 Nov 24;9:983992. doi: 10.3389/fmed.2022.983992 (PMC9729551; doi:10.3389/fmed.2022.983992)
Supplement: Supplementary file 1 [file Data_Sheet_1.pdf]

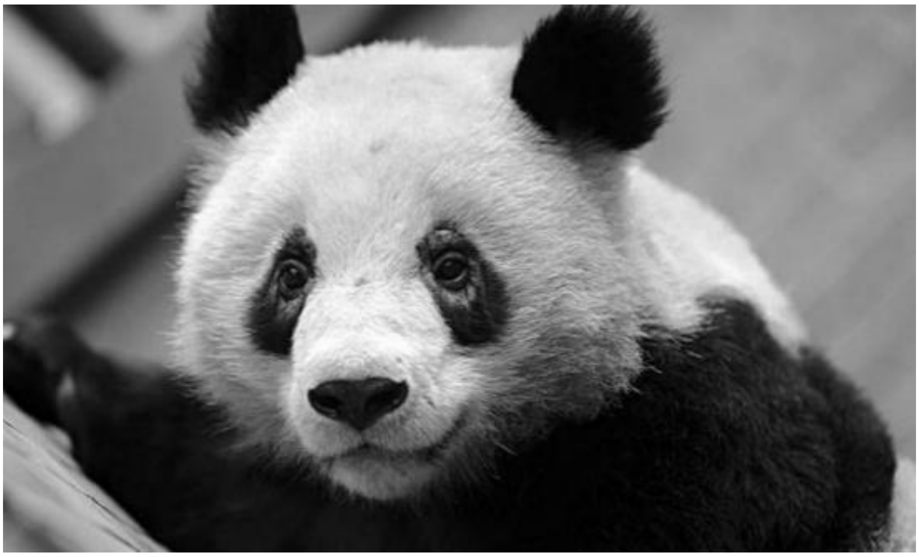

Supplementary Figure 1. The black eye circles of a giant panda that were turned to white

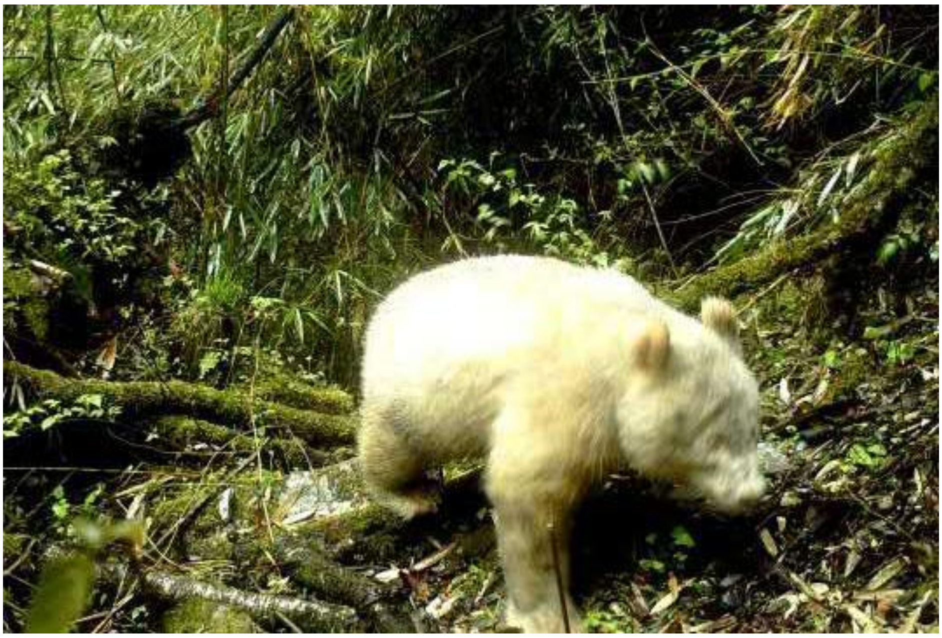

Supplementary Figure 2. A wild giant panda with albino phenotype

Supplementary Table 1.Sample information

Sample name	Gender	Age	Position
WGP_b1	Male	15	Back
WGP_b2	Female	11	Shoulder
WGP_w1	Male	15	Elbow
WGP_w2	Female	11	Shoulder

Supplementary Table 2.Sample sequencing output data quality assessment form

Sample name	Raw reads	Clean reads	Clean bases	Error rate(%)	Q20(%)	Q30(%)	GC content(%)
WGP_b1	53683504	52884216	7.93G	0.03	96.58	91.53	50.64
WGP_b2	52500110	51551574	7.73G	0.03	95.51	89.01	51.50
WGP_w1	52179014	51269284	7.69G	0.03	95.57	89.09	51.20
WGP_w2	51958048	50976064	7.65G	0.03	95.67	89.26	50.79

Supplementary Table 3.List of Reads vs. reference genome comparisons

Sample_name	WGP_b1	WGP_b2	WGP_w1	WGP_w2
Total reads	52884216	51551574	51269284	50976064
Total mapped	40259575 (76.13%)	46204260 (89.63%)	45944788 (89.61%)	45702721 (89.66%)
Multiple mapped	2011641 (3.8%)	1726449 (3.35%)	1421270 (2.77%)	1757599 (3.45%)
Uniquely mapped	38247934 (72.32%)	44477811 (86.28%)	44523518 (86.84%)	43945122 (86.21%)
Reads map to '+'	19097338 (36.11%)	22204644 (43.07%)	22236691 (43.37%)	21924363 (43.01%)
Reads map to '-'	19150596 (36.21%)	22273167 (43.21%)	22286827 (43.47%)	22020759 (43.2%)
